# Sex-Related Differences in Adolescent Physical Fitness, Physical Activity Levels and Enjoyment: A Two-Year Follow-Up of Italian Middle School Students

**DOI:** 10.3390/children13040484

**Published:** 2026-03-30

**Authors:** Alessandro Cudicio, Alice Iannaccone, Nicola Lovecchio, Claudio Orizio, Giacomo Smorgoni, Silvia Sangalli, Antonio Borgogni, Valeria Agosti

**Affiliations:** 1 Department of Human and Social Sciences, University of Bergamo, 25129 Bergamo, Italy; nicola.lovecchio@unibg.it (N.L.); silvia.sangalli@unibg.it (S.S.); antonio.borgogni@unibg.it (A.B.); 2 Department of Human Sciences, Society and Health, University of Cassino and Southern Lazio, 03043 Cassino, Italy; alice.iannaccone@unicas.it; 3 Department of Clinical and Experimental Sciences, Università degli Studi di Brescia, 25123 Brescia, Italy; claudio.orizio@unibs.it; 4 Department of Humanities, Philosophy and Education, University of Salerno, 84084 Fisciano, Italy; vaagosti@unisa.it

**Keywords:** early adolescence, physical activity, physical fitness, sex differences, enjoyment

## Abstract

**Highlights:**

**What are the main findings?**

- This research highlights a sex-specific pattern in body composition changes in middle school students over two years: the percentage of FM decreased significantly in males while increasing significantly in females. Moreover, unlike females, males improved their cardiorespiratory fitness and showed an increase in muscle mass. Regarding flexibility, while females remained stable, a decrease was observed in males.
- The analysis revealed a significant positive correlation between changes in MVPA and PA enjoyment for the whole sample; more specifically, the correlation was significant for males and not for females.

**What are the implications of the main findings?**

- In order to consider the fundamental biological sex distinctions and developmental differences, PE programs may need to be modulated, adapted and personalized to better address the sex-specific developmental needs of students.
- Because enjoyment is a key driver of physical activity and a protective factor against sport dropout—especially among girls aged 11–13—the observed increases in MPA and MVPA underline the important role of school PE in fostering active habits among both boys and girls.

**Abstract:**

Background: This two-year longitudinal study examined sex-specific changes and interrelations among body composition, physical fitness, physical activity (PA) enjoyment and PA levels during early adolescence. Methods: A cohort of 266 Italian middle school students (boys: n = 139; girls: n = 127) was assessed at two time points across two consecutive school years using anthropometry, field-based fitness tests, the Physical Activity Enjoyment Scale Questionnaire (Italian version) and a PA frequency questionnaire. Repeated-measures ANOVAs were used to explore time and time × sex effects, and correlations between change scores were calculated to explore associations between variables. Results: BMI increased in both sexes, while fat mass decreased in boys and increased in girls. Boys showed greater gains in cardiorespiratory fitness and muscular strength, whereas girls maintained better flexibility. Moderate PA and overall moderate-to-vigorous PA (MVPA) increased over time, with stronger positive associations between changes in PA enjoyment and MVPA in boys. Conclusion: These findings describe sex-specific patterns in physical fitness, body composition, and physical activity during early adolescence, and may help inform future research on sex-sensitive, individualized physical education approaches.

## 1. Introduction

The promotion of physical activity (PA) in childhood and adolescence is widely acknowledged as a public health and educational priority given its well-established contribution to physical, psychological, and social well-being and its role in the prevention of non-communicable diseases [1,2]. These developmental stages are also crucial for the education of long-term health-related behaviors. Evidence indicates that PA habits and fitness levels established early in life tend to persist into adulthood, highlighting the importance of promoting active lifestyles from a young age [3,4,5].

Nevertheless, international surveillance data reveal that the majority of children and adolescents do not meet the minimum recommended 60 min of moderate-to-vigorous daily PA [2], with significant declines observed across adolescence [6,7]. This decline is particularly marked during the transition from primary to middle school, a phase characterized by substantial developmental, social, and habit-related changes that may challenge the continuity of active behaviors [8,9].

Early adolescence is a period of profound physical and biological change, characterized by the onset of puberty [10]. This phase is marked by significant growth spurts and alterations in body composition, with distinct patterns emerging between the sexes. Males typically experience a greater increase in lean body mass and muscular strength, while females tend to have a greater accumulation of fat mass (FM) [11]. These sex-specific divergences in body composition are accompanied by differences in the development of physical fitness components, such as cardiorespiratory endurance, muscular fitness, and flexibility [12]. Critically, these physiological changes may also influence adolescents’ perceptions of their own bodies, as well as their self-efficacy and enjoyment of PA, which in turn can affect their overall PA level.

In light of these physiological and psychosocial changes, physical education (PE) emerges as a crucial, and often mandatory, component of the school curriculum. Thus, PE teachers play an important role in promoting teaching content related to active lifestyles. An effective and inclusive PE curriculum may benefit from moving beyond ‘one-size-fits-all’ classes toward approaches that consider developmental trends and sex-related differences. By incorporating regular physical evaluations and fitness assessments, educators may gain objective data that could help inform curricular content, set realistic and achievable goals, and modulate activities that are both appropriate and engaging for all students. A tailored approach may help improve physical fitness and may also foster a sense of enjoyment and self-efficacy, which are critical for motivating sustained PA habits throughout life [13,14].

Given the importance of the aspects outlined above, this study has three main objectives:

- To assess longitudinal changes in physical fitness, PA enjoyment, and overall PA levels over the first two years of middle school (11–13-year-old);
- To examine the sex-related differences in these variables;
- To investigate the associations between students’ individual changes in these variables.

## 2. Materials and Methods

### 2.1. Participants

A total of 266 students (139 males, 127 females) from seven schools in the northwest area of Italy participated in this two-year follow-up study. Students were evaluated in the first year of middle school, with an average age of 11.7 ± 0.42 years, and again in the second year, with an average age of 12.9 ± 0.42 years.

A power analysis was conducted using G*Power 3.1.9.7 for an ANOVA with repeated-measures, specifically for a within–between interaction effect. With an input effect size (f) of 0.115, an alpha error probability (α err prob) of 0.05, and a desired power (1 − β err prob) of 0.95, the analysis determined that a total sample size of 248 participants was required. This calculation was based on 2 groups, 2 measurements, and a correlation among repeated-measures of 0.5, with a nonsphericity correction (ϵ) of 1. The resulting actual power for this sample size was calculated as 0.9503138. Since our final sample size was 266, the study was adequately powered to detect small interaction effects.

All tests were conducted by a team of six Sport Science students during scheduled morning PE classes. Before data collection, all assessors underwent prior standardized training and followed the same measurement procedures in order to promote consistency and procedural standardization across schools and assessment waves.

The experimental design (comprensive of Ethical Committee approval) received approval from the Institutional Review Board of Regione Lombardia (Resolution of the Regional Council of 9 June 2017—n. X/6697) and the Italian National Olympic Committee (CONI). All aspects of the study were conducted in accordance with the World Medical Association Declaration of Helsinki [15], as revised in 2018.

### 2.2. Procedure

After explaining all procedures and the involved risks or discomforts, informed consent was obtained from all parents or legal guardians during the official enrollment, and verbal assent was obtained from the children prior to participation. After the anthropometric assessment, participants completed a battery of physical tests assessing cardiorespiratory fitness, muscular fitness, flexibility, and speed–agility. Subsequently, participants completed two questionnaires evaluating physical activity enjoyment and physical activity levels. No academic credits were given in case of participation. All the participants were free to withdraw from the study at any time. Participants who consented to participate in the study completed the physical fitness tests and questionnaires during a single school day (explained in detail later). One year later, they repeated the same protocol. During each assessment wave, anthropometric and body composition measures were collected first, followed by the physical fitness tests in a standardized order, and finally the classroom-based questionnaires on PA enjoyment and PA levels. Only data from participants who completed both data collection points are included in the present analysis.

### 2.3. Anthropometry and Body Composition Analysis

The measurements were conducted after participants removed their shoes, jackets, and any metallic accessories.

Height was measured to the nearest 0.5 cm using a stadiometer (seca213, Seca GmgH, Hamburg, Germany). Body mass was measured to the nearest 0.1 kg using a calibrated electronic scale (seca803, Seca GmgH, Hamburg, Germany). These values were used to calculate the body mass index (BMI) for each participant using the formula BMI = weight (kg)/height (m)^2^.

Body composition was assessed using a multi-frequency bioelectrical impedance analysis device (MF-HEXA, Akern S.r.l., Florence, Italy) as a practical field method. In the present study, body fat percentage (%FM) derived from the device output was used as an indirect field-based estimate of body composition. In pediatric populations, BIA-derived estimates depend on device-specific prediction equations, validation against reference methods, operating frequencies, and standardized measurement; accordingly, the present body composition values should be considered indirect field-based estimates [16,17]. Participants were required to rest in a supine position for a minimum of five minutes before the measurement to ensure fluid stabilization. Following the resting period, the skin surface on the dorsal side of the right hand and right foot was cleaned with an alcohol wipe. Four electrodes were then placed at standard anatomical locations on the hand and foot. General pre-assessment instructions were provided to reduce major sources of variability in BIA measurements; however, because testing was conducted in a school-based setting, pre-test conditions could not be strictly standardized or objectively verified for all participants. These precautions were adopted to reduce pre-analytical variability during data collection [17,18]. Biological maturation status (e.g., Tanner stage or maturity offset) was not assessed in this study.

### 2.4. Cardiorespiratory Fitness

Cardiovascular endurance was assessed using the Six-Minute walk test (6MWT) [19]. The test took place along a 20 m flat indoor corridor, with turnaround points clearly marked by cones. Due to school space constraints, we used a 20 m indoor corridor. This variation has been evaluated in pediatric populations [20,21]. Participants were instructed to walk as fast as possible, without running, for six minutes to cover the maximum distance. During the test, standardized phrases of encouragement (e.g., “You are doing well. Keep up the good work.”) were provided at the end of each minute. The test administrators counted the number of completed laps, and after six minutes, the total distance was calculated based on the number of laps plus the distance covered in the final partial lap and was recorded to the nearest meter.

### 2.5. Muscular Fitness

The standing broad jump test (SBJT) was used to assess lower limb explosive strength, following the protocol described by Ortega and colleagues [22]. Participants were instructed to stand behind a starting line with their feet parallel. From a stationary position and using a two-footed takeoff with free arm movement, they were instructed to jump for maximum horizontal distance. A jump was considered valid only if the participant landed with both feet simultaneously without falling down.

### 2.6. Flexibility

To assess lower back and hamstring flexibility, the V-sit and reach test (VSRT) was administered. A measuring tape was affixed to the floor, with a perpendicular line marking the zero point. To obtain only positive numbers, the zero point was set at 23 cm. Participants were seated barefoot on the floor, with their legs extended straight. The malleoli were separated by 30 cm with the measuring tape in the middle, and their heels placed directly on the zero-point line. Participants were instructed to place one hand over the other, palms down, and to exhale while slowly leaning forward, sliding their hands along the measuring tape as far as possible without bending their knees. The position of maximal reach had to be held for two seconds. The trial was not considered valid if the participant’s knees bent or if the hands separated. After one familiarization trial, each participant performed three valid trials, with a brief rest period between each. The score, representing the maximal distance reached from the zero line, was recorded to the nearest 0.5 cm. The highest of the three scores was used for subsequent data analysis [23].

### 2.7. Speed Agility

Agility, defined as the ability to rapidly change direction, was measured using the 4 × 10 m shuttle run test (SRT), as described in the HELENA study [22]. The course consisted of two parallel lines marked on a non-slip floor 10 m apart. From a standing start position behind one of the lines, participants ran as fast as possible to the opposite line upon a verbal command. They were required to touch the line with at least one foot before turning and running back to the starting line. This shuttle was repeated twice, for a total of four 10 m sprints covering a total distance of 40 m. The test was timed, with a digital chronometer system measuring in 0.01 s sensitivity (Casio HS-80TW-1, Tokyo, Japan), from the start command to the moment the participant’s torso crossed the finish line after completing the final shuttle.

Overall, previous methodological studies have reported acceptable reliability for field-based fitness tests in adolescent populations under comparable conditions, including the SBJT, flexibility testing, and the 4 × 10 m shuttle run [24], and test–retest reliability has also been documented for the 6MWT in healthy children [25].

### 2.8. PA Enjoyment

Participants’ enjoyment of PA was measured using the Physical Activity Enjoyment Scale (PACES-it), developed by Kendzierski and colleagues [26] and previously validated in Italian by Carraro [27]. The PACES-it is a 16-item self-report instrument that assesses the level of pleasure individuals derive from PA. Participants were asked to rate their agreement with each statement on a 5-point Likert-type scale, ranging from 1 (“Completely disagree”) to 5 (“Extremely agree”). In accordance with the scoring protocol, the negatively worded items were reverse-scored. The scores from all 16 items were then mediated to produce a total enjoyment score, with higher scores indicating a greater level of enjoyment in PA. The Italian version of the PACES has shown satisfactory psychometric reliability in Italian student samples [28].

### 2.9. Physical Activity Levels

The volume of daily PA was quantified using the previous day physical activity recall (PDPAR) questionnaire, a validated self-report instrument [29]. The questionnaire was administered in a classroom setting, on a day following a school day, where participants were guided by a researcher to systematically recall their activities from the previous day (from waking to bedtime).

The PDPAR worksheet was divided into 48 consecutive 30 min time blocks. For each block, participants identified their main activity and rated its intensity from very light to vigorous. To calculate a quantitative measure of PA, each reported activity was assigned a corresponding metabolic equivalent of task (MET) value based on the Compendium of Physical Activities [30]. The primary outcome variable was the total number of minutes spent daily in moderate PA (MPA), defined as any activity with a value of ≥3 METs and <6 METs; in vigorous PA (VPA), defined as ≥6 METs; and in moderate-to-vigorous PA (MVPA), defined as ≥3 METs. The PDPAR has previously shown acceptable reliability and validity in youth populations [29].

### 2.10. Data Analysis

Data were collected, cleaned and stored in an Excel (Microsoft Corporation, Redmond, DC, USA) table. Then, they were analyzed with Matlab R2025 A (MathWorks, Inc., Natick, MA, USA). To quantify the year-on-year change (Δ), the difference between the second-year and first-year results was calculated for each variable: BMI, %FM, 6MWT, SBJT, VSRT, SRT, PACES-it, moderate PA, vigorous PA, and MVPA.

### 2.11. Statistics Analysis

Descriptive statistics were calculated for all variables. Repeated-measures ANOVA was employed to evaluate longitudinal changes in physical fitness, PA enjoyment, and PA levels over two years, with sex included as a between-subjects factor. Assumptions of normality and sphericity for the repeated-measures ANOVA were checked, and when sphericity was violated, Greenhouse–Geisser-corrected degrees of freedom and *p* values were used. Post hoc comparisons were conducted using Tukey’s honestly significant difference test. Pearson correlation analysis was then used to examine relationships between the calculated change scores (Δ). Given the number of associations examined and the observational nature of the study, change-score correlation analyses were considered exploratory. No formal correction for multiple comparisons was applied to these analyses; therefore, the results, particularly smaller or marginal associations, should be interpreted cautiously. Repeated-measures ANOVA was chosen because the study included two assessment waves and the primary aim was to evaluate overall time, sex, and time x sex effects at the group level.

## 3. Results

A total of 266 students who completed both assessment waves were included in the analysis. Descriptive characteristics of the sample in the first and second years of middle school, stratified by sex, are reported in Table 1. As expected, body mass, height and BMI increased between the two school years in both boys and girls, reflecting normal growth during early adolescence. Performance in the physical fitness tests and levels of PA also differed between boys and girls, with boys generally showing higher values in cardiorespiratory fitness and muscular strength tests, and girls showing slightly better performance in flexibility (Table 1).

### 3.1. Anthropometry and Body Composition Analysis

**Figure 1:**
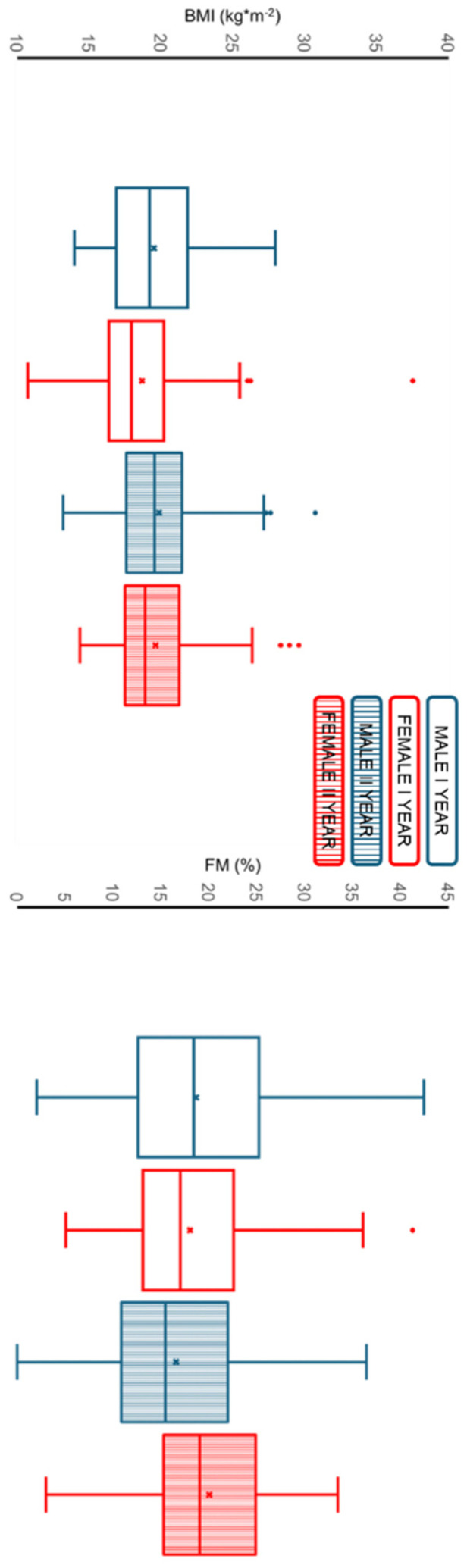
Sex-specific changes in anthropometric and body composition variables between the first and second years of middle school. Box plots show the distribution of BMI and FM in males and females at both time points. BMI: body mass index; FM: fat mass. Box plot legend: blue indicates male, red indicates female, empty boxes indicate year I, and filled boxes indicate year II.

The BMI (Figure 1) shows a significant main effect of time (the within-subjects factor) (*p* < 0.001; F = 38.42; η^2^p = 0.127) and no significant effect of sex (the between-subjects factor) (*p* = 0.178; F = 1.82; η^2^p = 0.007). A significant small interaction effect was observed between changes in BMI over time and sex (*p* = 0.005; F = 7.96; η^2^p = 0.029).

The FM (Figure 1) shows no significant effect of time (*p* = 0.906; F = 0.014; η^2^p < 0.001) and no significant effect of sex (*p* = 0.120; F = 2.43; η^2^p = 0.009). On the other hand, a significant interaction effect was observed between changes in FM over time and sex (*p* < 0.001; F = 32.98; η^2^p = 0.112). The post hoc test shows a significant decrease in males (*p* < 0.001) and a significant increase in females (*p* < 0.001).

### 3.2. Physical Fitness Tests

#### 3.2.1. Cardiorespiratory Fitness

**Figure 2:**
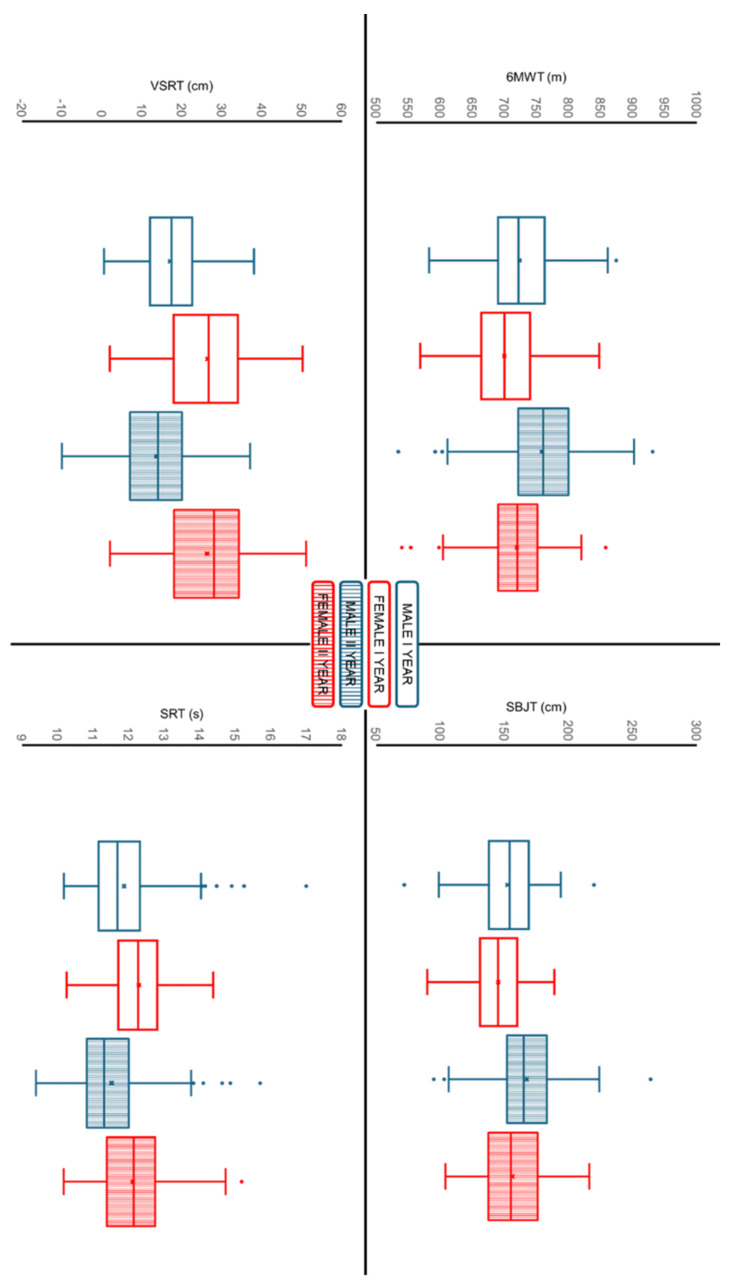
Sex-specific changes in physical fitness performance between the first and second year of middle school. Box plots show the distribution of 6MWT, SBJT, VSRT and SRT scores in males and females at both time points. 6MWT: six-minute walking test; SBJT: standing broad jump test; VSRT: V-sit and reach test; SRT: 4 × 10 m shuttle run test. Box plot legend: blue indicates male, red indicates female, empty boxes indicate year I, and filled boxes indicate year II.

The 6MWT (Figure 2) shows a significant main effect of time (*p* < 0.001; F = 88.48; η^2^p = 0.252) and a significant main effect of sex (*p* < 0.001; F = 25.7; η^2^p = 0.089). A significant small interaction effect was observed between changes in 6MWT over time and sex (*p* = 0.007; F = 7.42; η^2^p = 0.027).

#### 3.2.2. Muscular Fitness

The SBJT (Figure 2) shows a significant main effect of time (the within-subjects factor) (*p* < 0.001; F = 161.7; η^2^p = 0.380) and a significant main effect of sex (*p* < 0.001; F = 11.9; η^2^p = 0.043). No significant interaction effect was observed between changes in the SBJT over time and sex (*p* = 0.053; F = 3.79; η^2^p = 0.014).

#### 3.2.3. Flexibility

The VSRT (Figure 2) shows a significant main effect of time (*p* < 0.001; F = 16.15; η^2^p = 0.061) and a significant main effect of sex (*p* < 0.001; F = 106; η^2^p = 0.292). A significant small interaction effect was observed between changes in the VSRT over time and sex (*p* < 0.001; F = 12.8; η^2^p = 0.048). The post hoc test shows a significant decrease in males (*p* < 0.001) and no significant changes in females (*p* = 0.987).

#### 3.2.4. Speed Agility

The SRT (Figure 2) shows a significant main effect of time (*p* < 0.001; F = 27.3; η^2^p = 0.094) and a significant main effect of sex (*p* < 0.001; F = 20.7; η^2^p = 0.073). No significant interaction effect was observed between changes in the SRT over time and sex (*p* = 0.077; F = 3.15; η^2^p = 0.012).

### 3.3. Physical Activity Enjoyment Levels

#### 3.3.1. PA Enjoyment

**Figure 3:**
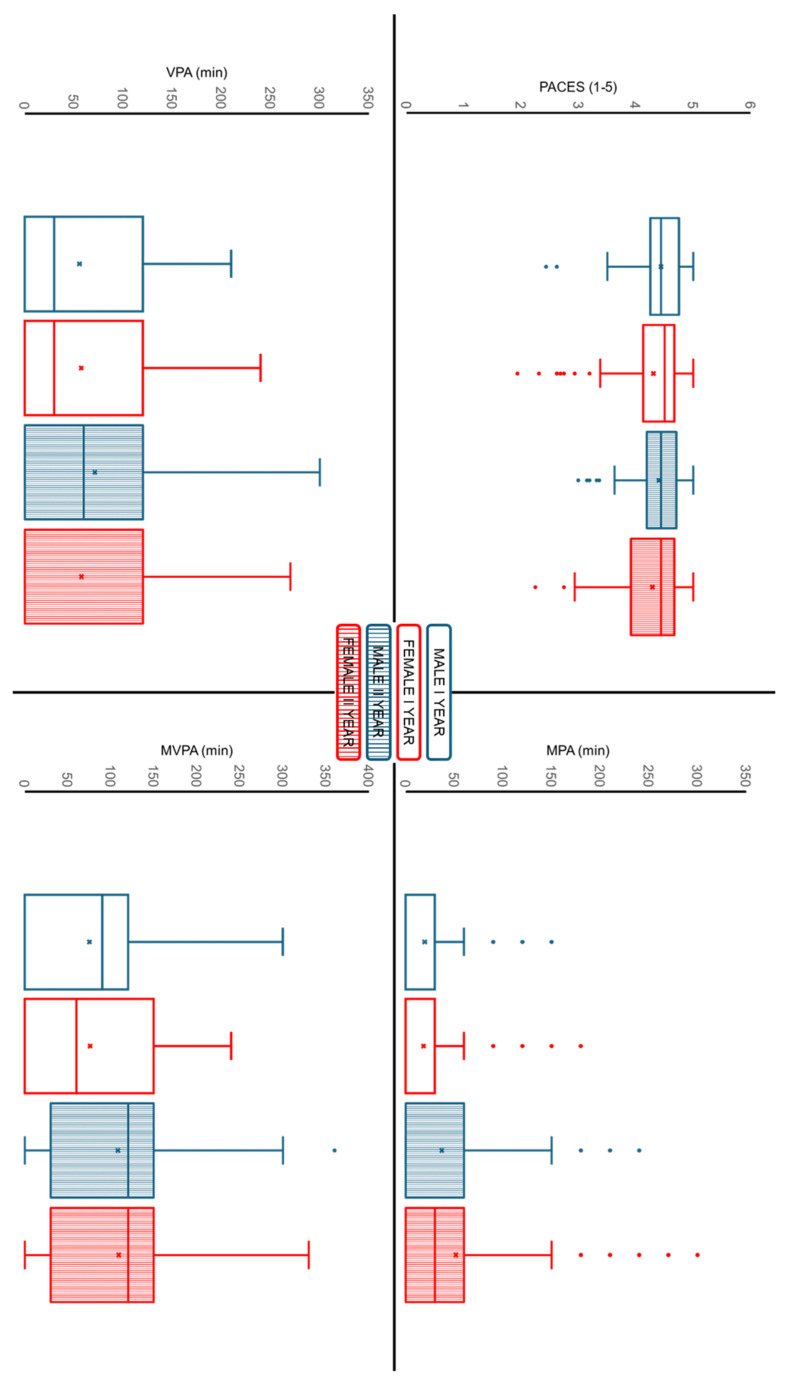
Sex-specific changes in PA enjoyment and PA levels between the first and second years of middle school. Box plots show the distribution of PACES-it scores and daily minutes of MPA, VPA and MVPA in males and females at both time points. PACES-it: Italian version of the physical activity enjoyment scale; MPA: moderate physical activity; VPA: vigorous physical activity; MVPA: moderate-to-vigorous physical activity. Box plot legend: blue indicates male, red indicates female, empty boxes indicate year I, and filled boxes indicate year II.

The PACES-it (Figure 3) shows no significant effect of time (*p* = 0.564; F = 0.333; η^2^p = 0.001) and a significant small main effect of sex (*p* = 0.033; F = 4.6; η^2^p = 0.019). No significant interaction effect was observed between changes in PACES-it over time and sex (*p* = 0.494; F = 0.469; η^2^p = 0.002).

#### 3.3.2. Physical Activity Levels

MPA (Figure 3) shows a significant main effect of time (*p* < 0.001; F = 38.07; η^2^p = 0.126) and no effect of sex (*p* = 0.153; F = 2.06; η^2^p = 0.008). No significant interaction effect was observed between changes in MPA over time and sex (*p* = 0.054; F = 3.75; η^2^p = 0.014).

VPA (Figure 3) shows no significant effect of time (*p* = 0.126; F = 2.36; η^2^p = 0.009) and no significant effect of sex (*p* = 0.373; F = 0.796; η^2^p = 0.003). No significant interaction effect was observed between changes in VPA over time and sex (*p* = 0.126; F = 2.36; η^2^p = 0.009).

MVPA (Figure 3) shows a significant main effect of time (*p* < 0.001; F = 34.2; η^2^p = 0.115) and no significant effect of sex (*p* = 0.903; F = 0.0148; η^2^p < 0.001). No interaction effect was observed between changes in MVPA over time and sex (*p* = 0.981; F = 0.0005; η^2^p < 0.001).

It should be noted that variability in self-reported PA was high, as reflected by large standard deviations, suggesting substantial inter-individual differences.

### 3.4. Change Scores

The Pearson’s correlations for the Δs of all the tested parameters were reported for the whole sample (Table 2), for males (Table 3) and for females (Table 4).

## 4. Discussion

This study aimed to longitudinally assess changes in physical fitness, PA enjoyment, and PA levels in middle school students over two years to explore sex-related differences and to correlate changes in these variables.

The findings of this study indicate significant longitudinal improvement in several physical fitness parameters, including cardiorespiratory fitness, muscular fitness, and speed agility, alongside notable sex-related differences, particularly in body composition and flexibility. While moderate PA showed an increase, overall PA enjoyment remained stable over the two-year period. Interestingly, positive associations were observed between changes in PA enjoyment and changes in MVPA, particularly in males.

### 4.1. Longitudinal Changes in Physical Fitness and Body Composition

#### 4.1.1. BMI and %FM

The observed increase in BMI over the two-year period for the entire sample aligns with general growth trends documented during early adolescence [31]. However, our analysis revealed a distinct, sex-specific pattern in %FM-derived body composition estimates. While BMI increased for both males and females, their percentage of FM followed divergent trajectories: it decreased significantly in males while increasing significantly in females. These findings are consistent with known sex-related developmental patterns during early adolescence [32,33,34,35,36]. This divergence is consistent with known pubertal patterns; however, in the absence of direct assessment of maturation status, differences in maturation timing may also have contributed [37]. These findings may be relevant when reflecting on how PE activities are adapted to students’ developmental characteristics during this period; however, the present observational design does not allow specific pedagogical recommendations to be drawn.

#### 4.1.2. Cardiorespiratory Fitness (6MWT)

The significant increase in 6MWT performance observed over the two-year study period is a positive and expected finding. This improvement in cardiorespiratory fitness aligns with the increased growth and height typically experienced during early adolescence [11]. The significant sex difference, with males demonstrating a greater improvement than females, is also a common finding in this age group [38]. This divergence may be attributed to a combination of factors, including hormonal changes that lead to greater muscle mass in males and a higher relative FM gain in females [11], although the absence of direct assessment of biological maturation prevents a more precise interpretation. Indeed, as can be seen in Table 2, the Δ 6MWT has an inverse correlation with the Δ %FM. This association may partly contribute to the observed interaction between changes in 6MWT and sex, but it should be interpreted cautiously. It is possible that the observed increase in MVPA was associated with these improvements in cardiorespiratory fitness; however, the direction of this relationship cannot be determined.

From a practical, biomechanical standpoint, the observed increase in height is directly linked to longer lower limbs, which in turn permits a greater stride length. This kinematic advantage allows an individual to cover more distance with each step, logically translating into improved functional performance and a greater total distance covered during the 6MWT [39].

#### 4.1.3. Muscular Fitness (SBJT)

The significant increase in SBJT performance over time for both sexes is consistent with the natural musculoskeletal development that occurs during early adolescence [38]. This period is characterized by rapid increases in muscle mass and strength, particularly in the lower limbs, which directly contributes to greater explosive strength as measured by the SBJT. The absence of a significant interaction effect over time and sex, despite a significant difference in performance between males and females, suggests that while boys and girls start with different average levels of strength, both groups follow a similar growth trajectory in terms of year-over-year gains. This finding aligns with the literature on musculoskeletal maturation during puberty, where strength gains are similar in both sexes [11,34,35,36] and where height and weight changes must be considered together, as their relationship is non-linear.

#### 4.1.4. Flexibility (VSRT)

Our finding of a significant difference over time in flexibility, specifically a decrease for males and no significant change for females, is a notable outcome. This sex-specific divergence in flexibility is well-documented in the literature and can be explained by the asynchrony between bone growth and soft tissue (muscles and tendons) lengthening during the male pubertal growth spurt [40]. As bones lengthen rapidly, the muscles and tendons may not stretch at the same rate, leading to a temporary reduction in flexibility. The significant sex difference and interaction we observed may be relevant for understanding flexibility changes during early adolescence; however, any practical implications for PE programming should be tested in dedicated intervention studies.

#### 4.1.5. Speed Agility (SRT)

The significant decrease in the SRT time observed over the two-year period is a positive finding, indicating a clear improvement in agility for the entire sample. While there was a significant difference between sexes, with males completing the test in less time than females, the lack of a significant interaction effect indicates that both boys and girls experienced a similar rate of improvement in their speed agility over the two-year period [41]. This suggests that middle school years may represent a period of overall positive development in this fitness component for both sexes, although the present study’s design does not allow the specific contribution of PE or other contextual factors to be determined [42].

#### 4.1.6. Longitudinal Changes: Summary

The changes in physical fitness observed in this study present a dual portrait. The first is a continuation of existing sex-based differences in strength and speed agility, with both males and females continuing to improve. The second, and perhaps more crucial, is the emergence of significant differentiation in %FM-derived body composition estimates, cardiorespiratory fitness, and flexibility during this period. The distinct dynamic of change between males and females in these areas suggests that developmental differences should be taken into account when interpreting adolescent fitness and %FM trajectories. These findings may help inform future research on how PE activities can be better aligned with students’ individual and developmental characteristics.

### 4.2. Physical Activity Enjoyment Levels

#### 4.2.1. Physical Activity Enjoyment

The lack of significant longitudinal change in the PACES-it over the two-year period is a crucial finding, as enjoyment is consistently identified as one of the strongest predictors of long-term PA adherence [43]. This stability in enjoyment, particularly in a developmental stage characterized by a general decline in PA participation, may be noteworthy; however, the present study did not directly assess environmental determinants such as PE curriculum characteristics, and therefore, no specific protective effect can be inferred [44]. While overall enjoyment remained stable, a significant sex difference was observed, with males reporting higher levels of enjoyment than females. Furthermore, the positive correlation between changes in VPA and PA enjoyment was significant for males but not for females, suggesting a stronger association between enjoyment and high-intensity activity in boys than in girls in this age group, although the direction of this relationship cannot be determined and bidirectionality is possible. These findings may support further reflection on sex-sensitive approaches in PE that take students’ competence and enjoyment into account. However, the present results do not allow causal conclusions about the role of enjoyment in shaping long-term PA habits. An oblique approach (i.e., differentiated and flexible instruction) may be a useful pedagogical perspective to explore in future applied research [45].

#### 4.2.2. Physical Activity Levels

Our finding of a significant increase in MPA and MVPA over the two-year period is a positive but somewhat counterintuitive result. The existing literature often documents a decline in PA during the transition to middle school [46]. The observed increase in our sample, with no significant differences between the sexes, may reflect contextual factors, including characteristics of the school environment, although these were not directly assessed. This could possibly reflect a favorable school and community environment (e.g., a structured PE curriculum and access to extracurricular sports), although we did not directly assess these factors. The fact that VPA did not show a significant change, while MPA and MVPA did, indicates that the increase in activity was primarily driven by moderate-intensity activities. This pattern may be relevant from a school-health perspective; however, no direct inference can be made regarding the specific contribution of school-based programs or PE. Although several studies report declines in PA during early adolescence, the increase observed in our sample may reflect contextual factors (e.g., school environment, measurement characteristics) and should therefore be interpreted cautiously. Moreover, given the reliance on a single-day self-report recall instrument, changes in MVPA may partly reflect reporting variability rather than true behavioral shifts [47].

### 4.3. Change Scores

A key objective of this study was to investigate the interrelationships between the observed longitudinal changes. The exploratory Pearson’s correlation analysis of the change scores (Δ) revealed several associations that should be interpreted cautiously, particularly when examining the data for the whole sample and for each sex independently.

In the whole sample (Table 2), a strong positive correlation was found between changes in BMI and FM (Δ BMI and Δ %FM, r = 0.485, *p* < 0.001). This expected result indicates that increases in overall weight are significantly associated with increases in body fat, supporting the value of considering both metrics together during this developmental period. Conversely, a significant negative correlation was found between changes in cardiorespiratory fitness and FM (Δ 6MWT and Δ %FM, r = -0.214, *p* < 0.001). This indicates an association between changes in cardiorespiratory endurance and changes in %FM; however, given the exploratory nature of the analysis and the number of comparisons performed, no mechanistic or causal interpretation should be drawn. This finding may also partly reflect the greater decrease in %FM and the larger improvement in cardiorespiratory fitness observed in males. We also found a modest but significant positive correlation between changes in lower limb explosive strength and cardiorespiratory fitness (Δ SBJT and Δ 6MWT, r = 0.180, *p* = 0.003), suggesting a possible association between cardiorespiratory and muscular fitness.

The analysis also revealed a strong link between changes in PA and changes in PA enjoyment. As expected, changes in MVPA were highly correlated with changes in MPA and VPA, largely reflecting the mathematical composition of the MVPA variable (Δ MVPA and Δ MPA, r = 0.493, *p* < 0.001; Δ MVPA and Δ VPA, r = 0.711, *p* < 0.001). A significant positive correlation was also observed between changes in MVPA and PA enjoyment (Δ MVPA and Δ PACES-it, r = 0.166, *p* = 0.011), indicating a positive association between changes in enjoyment and overall PA levels.

A closer look at the sex-specific correlations (Table 3 and Table 4) provides further insight. The relationship between changes in VPA and PA enjoyment, while positive for the whole sample, shows a marked sex difference. For males, the correlation was significant (Δ VPA and Δ PACES-it, r = 0.230, *p* = 0.011), suggesting a stronger association between enjoyment and VPA in boys. In contrast, the correlation for females was not significant (Δ VPA and Δ PACES-it, r = 0.067, *p* = 0.474). A similar pattern was observed for MPA and MVPA, where the correlation with PA enjoyment was significant for males (Δ MPA and Δ PACES-it, r = 0.182, *p* = 0.045; Δ MVPA and Δ PACES-it, r = 0.324, *p* < 0.001) but not for females, suggesting a potentially sex-specific pattern in the observed association between PA and enjoyment. Another sex-specific finding was the correlation between changes in speed agility and muscular fitness for males (Δ SRT and Δ SBJT, r = −0.235, *p* = 0.005), which was not observed in females. In Italy, girls’ regular participation in PA, which is already lower than boys’, falls markedly between the ages of 11–13 and 14–17 (from 60.4% to 42.2%) [48]. PE teachers may play an important role in this context. The present findings may support the use of activities that are better aligned with students’ motor skill levels and interests, with the aim of fostering enjoyment and encouraging regular PA in daily life. However, specific educational strategies should be evaluated in dedicated intervention studies.

### 4.4. Limitations

Despite these strengths, the study has several limitations. Physical activity and enjoyment were assessed through self-report instruments, which may be influenced by recall and social desirability bias. In addition, although assessors received prior standardized training, no study-specific test–retest or inter-rater reliability coefficients were collected; therefore, the extent to which smaller longitudinal changes exceeded measurement error cannot be directly quantified. As also highlighted in recent methodological work, the interpretation of performance changes over time is strengthened when measurement procedures are clearly standardized and reliability-oriented [49]. The geographic specificity of the sample limits generalizability. Biological maturation was not directly assessed; given the age range of the participants, differences in pubertal timing may have influenced changes in %FM-derived body composition estimates and activity levels. This issue is particularly relevant for body composition estimates obtained by BIA in a school-based field setting, where measurement variability may be influenced by several procedural and pre-analytical factors (e.g., hydration status, recent food intake, and prior physical activity). In children and adolescents, the accuracy of BIA-derived estimates is influenced by growth-related physiological factors, hydration status, body position, operating frequency, and the use of device- and population-specific equations. Although general pre-assessment instructions were provided, pre-test factors known to influence BIA measurements were not strictly standardized or objectively monitored in the school setting; therefore, values obtained from different devices are not necessarily interchangeable for individual body fat estimation, and the present %FM findings should be interpreted with caution. In the present study, BIA was used primarily to characterize body composition change as % of FM. Accordingly, the body composition findings should be interpreted primarily as field-based estimates of longitudinal patterning rather than precise criterion measures of absolute body composition.

The use of only two measurement points provides a limited view of developmental trajectories. Although a repeated-measures ANOVA is appropriate for two assessment waves and for testing group-level time, sex, and time × sex effects, it does not capture individual variability and trajectories as flexibly as mixed-effects models. Change-score analyses were exploratory and involved multiple comparisons without formal correction; therefore, these findings should be interpreted cautiously, particularly in the case of small effect sizes or associations with *p* values close to the significance threshold. Finally, fitness measures were not adjusted using allometric scaling relative to body size during a period of rapid growth.

## 5. Conclusions

In conclusion, this two-year longitudinal study shows that the first two years of middle school are characterized by marked changes in %FM-derived body composition estimates, physical fitness and PA levels, with clear sex-specific trajectories. BMI increased in both sexes, while %FM decreased in boys and increased in girls, and boys outperformed girls in cardiorespiratory fitness and muscular strength, whereas girls maintained an advantage in flexibility. Moderate and overall MVPA increased over time, and changes in PA enjoyment were positively related to changes in MVPA, suggesting that enjoyment may be relevant for understanding PA-related patterns during early adolescence, particularly among boys. Overall, these results describe developmental patterns over time in fitness, PA enjoyment, and PA levels during early adolescence. Such patterns may help inform future research on interventions designed to account for sex-related and developmental differences. Future studies should include more frequent assessments, objective measures of PA, and designs specifically suited to testing school-based interventions. Overall, these findings may therefore support the development and future evaluation of sex-sensitive, individualized PE approaches that prioritize both competence and enjoyment.

## Figures and Tables

**Table 1:** Anthropometric characteristics, physical fitness measures, and physical activity levels of participants stratified by sex (male and female) and school year (year I and year II). Data are presented as mean ± standard deviation.

	Year I		Year II	
	Male	Female	Male	Female
*Anthropometry*				
Age (y)	11.7 ± 0.44	11.7 ± 0.41	12.9 ± 0.44	12.9 ± 0.41
Weight (kg)	44.4 ± 9.86	42.9 ± 8.82	50.8 ± 11.20	49.5 ± 9.05
Height (cm)	150 ± 7.9	151 ± 6.7	159 ± 8.65	159 ± 5.54
BMI (kg/m^2^)	19.5 ± 3.35	18.7 ± 3.49	19.9 ± 3.35	19.6 ± 3.13
FM (%)	18.7 ± 8.14	18.0 ± 7.10	16.7 ± 7.88	20.0 ± 6.32
*Physical fitness*				
6MWT (m)	724 ± 55.8	700 ± 51.2	759 ± 63.1	719 ± 52.8
SBJT (cm)	153 ± 22.4	145 ± 20.4	167 ± 25.8	156 ± 24.1
VSRT (cm)	17.1 ± 7.76	26.4 ± 10.50	13.6 ± 8.11	26.3 ± 11.80
SRT (s)	11.9 ± 1.09	12.3 ± 0.84	11.5 ± 1.05	12.1 ± 0.94
*Questionnaire*				
PACES-it (1–5)	4.44 ± 0.431	4.31 ± 0.563	4.40 ± 0.444	4.29 ± 0.515
MPA (min/day)	19.6 ± 35.90	18.4 ± 33.60	36.9 ± 53.70	51.5 ± 70.50
VPA (min/day)	55.7 ± 61.00	57.6 ± 66.00	71.2 ± 71.30	57.6 ± 70.20
MVPA (min/day)	75.3 ± 69.50	76.1 ± 66.10	108 ± 80.80	109 ± 79.30

BMI: body mass index; FM: fat mass; 6MWT: six-minute walking test; SBJT: standing broad jump test; VSRT: V-sit and reach test; SRT: 4 × 10 m shuttle run test; PACES-it: Italian version of the physical activity enjoyment scale; MPA: moderate physical activity; VPA: vigorous physical activity; MVPA: moderate-to-vigorous physical activity.

**Table 2:** Pearson’s correlation of Δs in the whole sample.

		∆ BMI	∆ %FM	∆ 6MWT	∆ SBJT	∆ VSRT	∆ SRT	∆ PACES-it	∆ MPA	∆ VPA
**∆ %FM**	r	**0.485**								
	*p*	<0.001 ***								
**∆ 6MWT**	r	−0.078	**−0.214**							
	*p*	0.206	<0.001 ***							
**∆ SBJT**	r	−0.019	−0.112	**0.180**						
	*p*	0.752	0.069	0.003 **						
**∆ VSRT**	r	**0.145**	0.014	−0.055	−0.001					
	*p*	0.018 *	0.821	0.369	0.988					
**∆ SRT**	r	0.116	**0.132**	0.058	**−0.203**	0.115				
	*p*	0.060	0.032 *	0.346	<0.001 ***	0.061				
**∆ PACES-it**	r	−0.004	0.061	0.063	−0.016	0.025	−0.109			
	*p*	0.952	0.350	0.337	0.808	0.697	0.096			
**∆ MPA**	r	0.045	0.090	−0.025	0.049	0.076	−0.028	0.036		
	*p*	0.467	0.144	0.680	0.425	0.216	0.648	0.580		
**∆ VPA**	r	−0.001	−0.026	0.068	−0.047	−0.075	0.007	**0.153**	**−0.262**	
	*p*	0.991	0.674	0.269	0.446	0.225	0.906	0.019 *	<0.001 ***	
**∆ MVPA**	r	0.032	0.042	0.043	−0.006	−0.012	−0.014	**0.166**	**0.493**	**0.711**
	*p*	0.603	0.493	0.490	0.916	0.849	0.821	0.011 *	<0.001 ***	<0.001 ***

Δ: difference between the second-year and first-year results; BMI: body mass index; %FM: percentage of fat mass; 6MWT: six-minute walking test; SBJT: standing broad jump test; VSRT: V-sit and reach test; SRT: 4 × 10 m shuttle run test; PACES-it: Italian version of the physical activity enjoyment scale; MPA: moderate physical activity; VPA: vigorous physical activity; MVPA: moderate-to-vigorous physical activity. Statistically significant results are reported in bold, * *p* < 0.05; ** *p* < 0.01; *** *p* < 0.001.

**Table 3:** Pearson’s correlation of Δs in the male sample.

		∆ BMI	∆ %FM	∆ 6MWT	∆ SBJT	∆ VSRT	∆ SRT	∆ PACES-it	∆ MPA	∆ VPA
**∆ %FM**	r	**0.525**								
	*p*	<0.001 ***								
**∆ 6MWT**	r	−0.158	**−0.216**							
	*p*	0.065	0.011 *							
**∆ SBJT**	r	−0.059	**−0.178**	0.138						
	*p*	0.487	0.036 *	0.107						
**∆ VSRT**	r	0.082	−0.054	−0.060	−0.105					
	*p*	0.335	0.528	0.486	0.217					
**∆ SRT**	r	0.121	0.134	0.104	**−0.235**	**0.191**				
	*p*	0.157	0.118	0.226	0.005 **	0.024 *				
**∆ PACES-it**	r	−0.073	0.004	0.098	−0.010	0.040	−0.083			
	*p*	0.423	0.961	0.288	0.909	0.664	0.364			
**∆ MPA**	r	0.012	0.069	−0.006	0.023	−0.049	−0.020	**0.182**		
	*p*	0.885	0.421	0.944	0.787	0.565	0.812	0.045 *		
**∆ VPA**	r	0.012	0.051	0.126	−0.071	−0.003	0.075	**0.230**	**−0.185**	
	*p*	0.893	0.551	0.140	0.409	0.967	0.381	0.011 *	0.029 *	
**∆ MVPA**	r	0.018	0.088	0.113	−0.052	−0.032	0.058	**0.324**	**0.411**	**0.820**
	*p*	0.834	0.306	0.187	0.544	0.709	0.501	<0.001 ***	<0.001 ***	<0.001 ***

Δ: difference between the second-year and first-year results; BMI: body mass index; %FM: percentage of fat mass; 6MWT: six-minute walking test; SBJT: standing broad jump test; VSRT: V-sit and reach test; SRT: 4 × 10 m shuttle run test; PACES-it: Italian version of the physical activity enjoyment scale; MPA: moderate physical activity; VPA: vigorous physical activity; MVPA: moderate-to-vigorous physical activity. Statistically significant results are reported in bold, * *p* < 0.05; ** *p* < 0.01; *** *p* < 0.001.

**Table 4:** Pearson’s correlation of Δs in the female sample.

		∆ BMI	∆ %FM	∆ 6MWT	∆ SBJT	∆ VSRT	∆ SRT	∆ PACES-it	∆ MPA	∆ VPA
**∆ %FM**	r	**0.375**								
	*p*	<0.001 ***								
**∆ 6MWT**	r	0.093	−0.094							
	*p*	0.300	0.291							
**∆ SBJT**	r	0.077	0.077	**0.200**						
	*p*	0.392	0.391	0.024 *						
**∆ VSRT**	r	0.143	−0.086	0.032	**0.177**					
	*p*	0.109	0.335	0.723	0.046 *					
**∆ SRT**	r	0.073	0.054	0.044	−0.138	−0.013				
	*p*	0.414	0.544	0.627	0.122	0.886				
**∆ PACES-it**	r	0.056	0.105	0.030	−0.014	−0.010	−0.153			
	*p*	0.552	0.262	0.754	0.884	0.913	0.102			
**∆ MPA**	r	0.036	0.044	−0.007	0.102	0.123	−0.060	−0.085		
	*p*	0.690	0.620	0.940	0.256	0.169	0.500	0.367		
**∆ VPA**	r	0.021	−0.058	−0.044	−0.044	−0.112	−0.054	0.067	**−0.320**	
	*p*	0.817	0.519	0.624	0.627	0.209	0.548	0.474	<0.001 ***	
**∆ MVPA**	r	0.048	−0.012	−0.044	0.049	0.008	−0.098	−0.014	**0.575**	**0.592**
	*p*	0.590	0.892	0.626	0.586	0.932	0.274	0.885	<0.001 ***	<0.001 ***

Δ: difference between the second-year and first-year results; BMI: body mass index; %FM: percentage of fat mass; 6MWT: six-minute walking test; SBJT: standing broad jump test; VSRT: V-sit and reach test; SRT: 4 × 10 m shuttle run test; PACES-it: Italian version of the physical activity enjoyment scale; MPA: moderate physical activity; VPA: vigorous physical activity; MVPA: moderate-to-vigorous physical activity. Statistically significant results are reported in bold, * *p* < 0.05; *** *p* < 0.001.

## Data Availability

All data supporting the findings of this study are available at https://doi.org/10.5281/zenodo.17877965.

## Abbreviations

The following abbreviations are used in this manuscript:

6MWT	Six-minute walk test
BMI	Body mass index
FM	Fat mass
MET	Metabolic equivalent of task
MPA	Moderate physical activity
MVPA	Moderate-to-vigorous physical activity
PA	Physical activity
PACES-it	Italian version of the physical activity enjoyment scale
PDPAR	Previous day physical activity recall
PE	Physical education
SBJT	Standing broad jump test
SRT	4 × 10 m shuttle run test
VPA	Vigorous physical activity
VSRT	V-sit and reach test
